# Case Report: *FAS* spontaneous mutation in a familial hemophagocytic lymphohistiocytosis patient with a complex heterozygous mutation in *PRF1*

**DOI:** 10.3389/fimmu.2025.1613433

**Published:** 2025-08-20

**Authors:** Wenwen Ding, Danni Li, Qi Yu, Yuping Wang, Lei Zhang

**Affiliations:** Department of Hematology, Qingdao Women and Children's Hospital, Qingdao, China

**Keywords:** *FAS* mutation, PRF1 mutation, autoimmune lymphoproliferative syndrome, hemophagocytic lymphohistiocytosis, comutation

## Abstract

Familial hemophagocytic lymphohistiocytosis type 2 (FHL2), caused by perforin 1 (PRF 1), is a rare and fatal autosomal recessive disorder characterized by a hyperinflammatory syndrome and the accumulation of activated T lymphocytes and histiocytes in the reticuloendothelial system. Autoimmune lymphoproliferative syndrome (ALPS) is an autoimmune disease that typically presents in children with lymphadenopathy, splenomegaly, and cytopenias or lymphomas. We report a case of a 9-year-old boy who was newly diagnosed with FHL, carrying a new type of compound heterozygous mutations (c.305G>T and c.139G>T) in *PRF1* and a spontaneous heterozygous mutation in the *FAS* gene (c.776T>C). He met six of the eight hemophagocytic lymphohistiocytosis (HLH) diagnostic criteria: fever, splenomegaly, cytopenia, hypofibrinogenemia, hemophagocytosis in the bone marrow, and elevated sCD25. He also had a high proportion of CD3^+^CD4^−^CD8^−^ T lymphocytes and a spontaneous mutation of *FAS*, which are features of ALPS. Chemotherapy failed to control the disease, and the child died within 3 months. *FAS* and *PRF1* comutation may have contributed to the adverse outcome in this patient. Notably, hematopoietic stem cell transplantation (HSCT) should be performed early in these patients.

## Introduction

Familial hemophagocytic lymphohistiocytosis (FHL) and autoimmune lymphoproliferative syndrome (ALPS) are diseases caused by defects in apoptotic pathways. They have overlapping clinical manifestations. The former is caused by defects in the cytotoxic granule pathway, and the latter is caused by defects in the Fas/FasL pathway. The accumulation of activated T lymphocytes is the common pathogenesis mechanism of HLH and ALPS. *PRF1* mutation causes familial hemophagocytic lymphohistiocytosis type 2 (FHL2), and *FAS* mutation causes ALPS.

Defects in the cytotoxic granule pathway have been reported to be highly correlated with the development of ALPS with defective *Fas* function (1–3). *PRF1* mutations have been reported to increase susceptibility to ALPS by 63-fold among patients with *FAS* mutations (3).

Whether *FAS* mutations influence the progression of HLH in patients with *PRF1* mutations has not been reported. Carriers of the same mutation may not ultimately develop FHL2. The existence of additional mechanisms that could synergize with defective *PRF1* function to promote the development of FHL2 remains unclear. Consequently, some scholars (4) have proposed a second-hit hypothesis, suggesting that a “second” mutation beyond the *PRF1* gene might represent a potentially critical mechanism for triggering the onset of FHL2.

Herein, we report an FHL2 patient with a spontaneous mutation in *FAS* and a complex heterozygous mutation in *PRF1*.

## Patient presentation

The patient presented with sustained fever and hepatosplenomegaly, and he was treated with antibiotics and a low dose of dexamethasone at a community clinic before being admitted to our department. He had palpable cervical lymphadenopathy, splenomegaly, and hepatomegaly. His laboratory indicators are shown in **Table 1**. We subsequently performed next-generation sequencing (NGS) on the patient’s blood to identify pathogenic microorganisms, including viruses, bacteria, fungi, and parasites; the results were negative. Antinuclear antibody and extractable nuclear antigen tests yielded negative results.

**Table 1:** Initial laboratory tests.

Laboratory tests	Initial laboratory test results	Laboratory test results after 3 days	Reference range
WBC	3.2 × 10^9^/L	2.12 × 10^9^/L	4–10 × 10^9^/L
NEU	0.88 × 10^9^/L	0.71 × 10^9^/L	1.7–7.7 × 10^9^/L
Platelets	58 × 10^9^/L	50 × 10^9^/L	100–300 × 10^9^/L
Hemoglobin	110 g/L	92 g/L	110–170 g/L
ALT	218 U/L	106 U/L	9–50 U/L
AST	143 U/L	77 U/L	15–40 U/L
Albumin	36 U/L	30 g/L	40–55 g/L
Fibrinogen	1.77 g/L	1.29 g/L	2–4 g/L
LDH	437 U/L	406 U/L	120–250 U/L
Ferritin	413 ng/ml	427 ng/ml	25–200 ng/ml
Peripheral blood smear	Atypical lymphocytes	–	Negative
Bone marrow aspiration	A small number of phagocytic cells	–	Negative
Antinuclear antibody and extractable nuclear antigens	–	Negative	Negative

The patient met five criteria for hemophagocytic lymphohistiocytosis (HLH): fever, splenomegaly, cytopenias, hypofibrinogenemia, and hemophagocytosis in the bone marrow, without EB virus infection. Methylprednisolone (10 mg/kg/day for 3 days) was administered, followed by a tapered dose of dexamethasone. Given that the levels of ferritin and hemophagocytes did not increase significantly, it was decided not to administer etoposide for chemotherapy. Moreover, additional laboratory tests were performed, and the results are shown in **Table 2**.

**Table 2:** Additional laboratory tests.

Laboratory tests	Laboratory test results	Reference range
Lymphocyte subpopulation		
CD3^+^CD4^−^CD8^−^ T cells	4.19%	0.82%–2.91%
NK cells	36.25%	10.01%–26.98%
Triglyceridemia	1.39 mmol/L	0–1.7 mmol/L
sCD25	32,868 pg/ml	410–2,623 pg/ml
Vitamin B12	562.9 pg/ml	140–701 pg/ml
Detection of toxic particles in the cytoplasm of NK cells		
Perforin	99.57%	> 78%
Granzyme B	94.01%	> 84%

We detected an abnormally elevated sCD25 level, which is often seen with HLH. Flow cytometry analysis of bone marrow cells revealed a group of NK cells (accounting for 3.1% of nuclear cells) with an abnormal phenotype (CD7^−^, CD2^+^, CD3^−^, CD5^−^, CD56^+^, CD94^+^, CD4^−^, CD8^dim−^, and CD16^dim+^). The expression of killer cell immunoglobulin-like receptor (KIR) antigens (CD158a, CD158b, CD158e, and CD158i) was extremely low. Notably, KIRs, also known as CD158, are expressed stochastically on the surface of NK cells. Limited or negative expression of KIRs may suggest NK-cell clonality. Moreover, malignant tumors, especially lymphoma, may be associated with HLH. Therefore, the patient underwent positron emission tomography–computed tomography (PET-CT) and cervical lymph node biopsy. PET-CT showed multiple enlarged lymph nodes. Cervical lymph node biopsy revealed reactive hyperplasia of the lymph tissue, and no clonal cell populations were identified.

**Figure 1:**
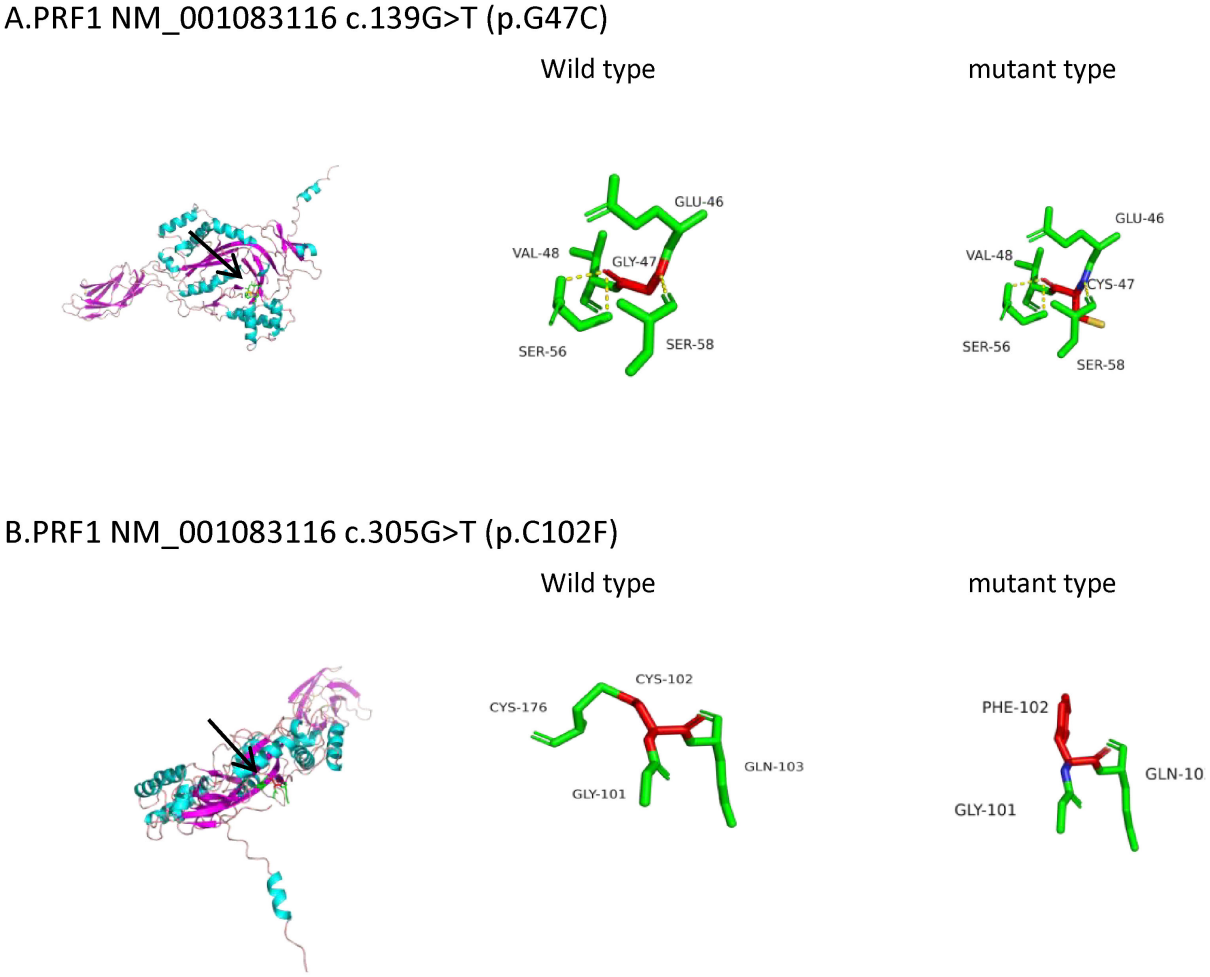
Three-dimensional (3D) structures of the *PRF1* protein. *PRF1* gene sequences (NM_001083116) were obtained from NCBI (https://www.ncbi.nlm.nih.gov/). The 3D protein structures of the *PRF1* wild type were generated using Swiss-Model (https://swissmodel.ExPASy.org/interactive). The A0A7J7FIP3.1.A template was used for both the *PRF1* wild and mutant types. The 3D protein structure was visualized using PyMOL 2.5. The arrow points to the location where the mutations lie. **(A)** *PRF1*:c.139G>T (p.G47C). The mutation is located in the beta fold. The 47th amino acid of wild-type perforin is glycine, which forms three hydrogen bonds with the two adjacent serines and disulfide bonds with the adjacent glutamate and valine. c.139G>T (p.G47C) causes glycine to be replaced by cysteine, which still forms three hydrogen bonds with the two adjacent serines and connects to glutamic acid and valine via disulfide bonds. The hydrogen bond and the disulfide bond have not changed. Protein stability may not have changed. **(B)** *PRF1*:c.305G>T (p.C102F). The mutation is located in the loop structure. The 102nd amino acid of wild-type perforin is cysteine, which is connected to the adjacent glycine, glutamine, and cysteine by disulfide bonds. c.305G>T (p.C102F) causes the cysteine to be replaced by phenylalanine and still connects to glycine and glutamine with disulfide bonds, losing the disulfide bond formed with cysteine. Disulfide bonds play a role in protein folding and stability. The reduction of disulfide bonds may affect the stability of the protein structure.

**Figure 2:**
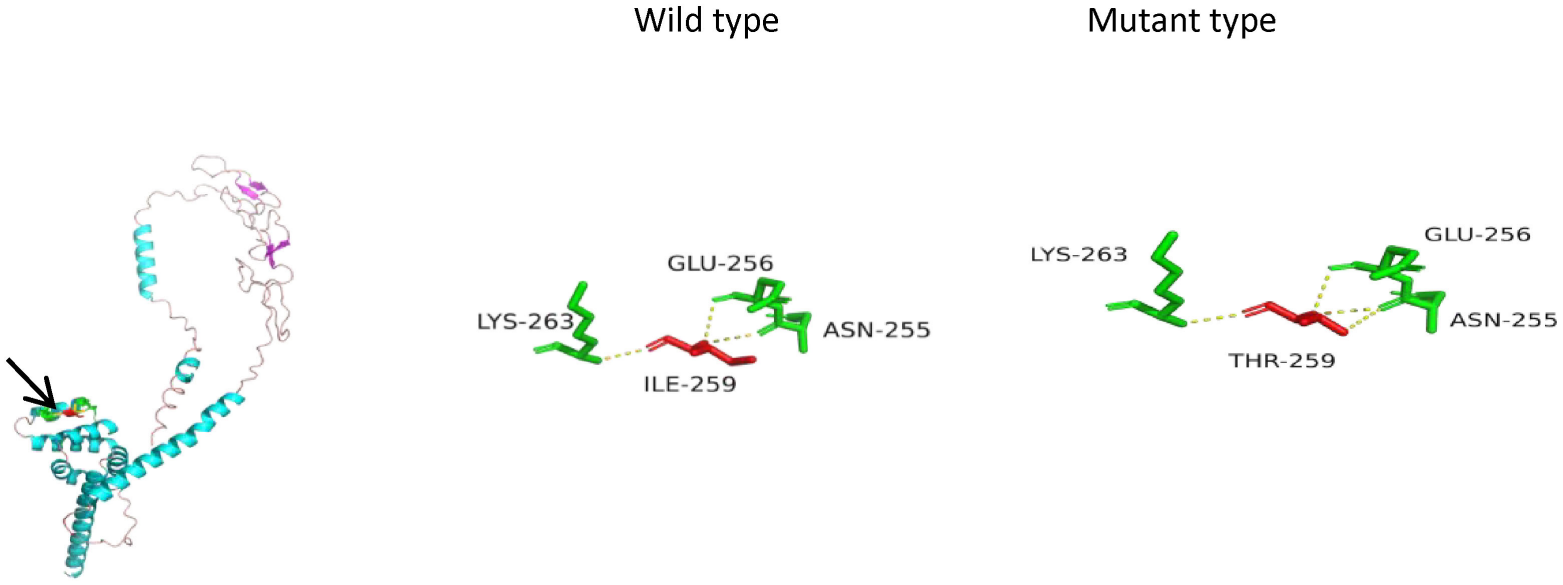
Three-dimensional (3D) structures of the FAS protein. FAS gene sequences (NM_000043) were obtained from NCBI (https://www.ncbi.nlm.nih.gov/). The 3D structures of the FAS wild type were generated using Swiss-Model (https://swissmodel.ExPASy.org/interactive). P25445.1.A was used as the template for both the *FAS* wild type and mutant type. The 3D protein structure was generated using PyMOL 2.5. The arrow points to the location where the mutation lies. In the mutant type, isoleucine at position 259 was replaced by threonine. The number of hydrogen bonds changed from three to four (yellow dashed line), which suggests a reduction in protein stability.

His fever was briefly controlled, with liver and spleen retraction. However, he developed a fever again approximately 2 weeks later. Subsequently, we obtained the results of the genomic analysis, which revealed the following: two rare heterozygous missense variations (c.139G>T, p.G47C; c.305G>T, p.C102F) in the *PRF1* gene and a spontaneous heterozygous mutation in the *FAS* gene (c.776T>C, p.I259T). Testing of samples from the immediate family revealed that the mother carried a *PRF1*:c.139G>T heterozygous mutation, and the father had a c.305G>T mutation. His elder sister also carried the *PRF1*:c.139G>T mutation. They were healthy. The variants were classified as likely pathogenic according to the American College of Medical Genetics and Genomics (ACMG). The *FAS* mutation was a spontaneous missense mutation and was classified as pathogenic according to the ACMG. The predicted structures of the mutant perforin and FAS proteins (**Figures 1**, **2**) were generated using PyMOL 2.5. The results showed that the p.C102F variant led to the loss of disulfide bonds and might affect the stability of the perforin protein. The p.I259T variant altered the number of hydrogen bonds and might affect the stability of the FAS protein. These three missense mutations were predicted to be deleterious by Polyphen-2, REVEL, SIFT, MutationTaster, and GERP^+^, and the scores are shown in **Table 3**.

**Table 3:** The scores for the harmfulness of these three gene mutations using gene mutation predicton softwares.

Gene mutation prediction software	Scores		
	*PRF1*:c.139G>T	*PRF1*:c.305G>T	*FAS*:c.776T>C
Polyphen-2	1 (probably damaging)	0.997 (probably damaging)	1 (probably damaging)
REVEL	0.887 (D)	0.828 (D)	0.875 (D)
SIFT	0 (damaging)	0 (damaging)	0.001 (damaging)
MutationTaster	1 (disease-causing)	1 (disease-causing)	1 (disease-causing)
GERP^+^	5.7 (conserved)	5.65 (conserved)	4.65 (conserved)

According to the HLH-2004-based diagnostic criteria (5), the patient met the diagnosis of FHL2 and had no indicators of secondary HLH, such as malignancies, infections (especially Epstein–Barr virus), or rheumatoid disorders. Moreover, multiple lymphadenopathies, hepatosplenomegaly, cytopenias, an increased proportion of CD3^+^CD4^−^CD8^−^ T lymphocytes, and a spontaneous mutation of the *FAS* gene supported the diagnosis of ALPS. Notably, we did not further divide the cells into TCRαβ^+^ cells (known as DNTs) and TCRγδ^+^ cells, as the latter type is primarily nonspecifically elevated in other diseases, such as EBV-associated lymphocyte proliferation (6).

The patient was transferred to another hospital, where he underwent chemotherapy and rapamycin treatment while awaiting allogeneic hematopoietic stem cell transplantation. However, the disease remained uncontrolled, and the child eventually died of HLH within a few months.

## Discussion

In the present case report, we described the phenotype of a 9-year-old boy who carried compound heterozygous mutations (c.139G>T, p.G47C; c.305G>T, p.C102F) in *PRF1* and a spontaneous mutation (c.776T>C, p.I259T) in the *FAS*, presenting with features of FHL and ALPS.

*PRF1* gene mutation accounts for 20%–40% of FHL patients and is termed FHL2 (7, 8). Variants of *FAS* are usually related to ALPS type 1A (ALPS 1a). Both are characterized by lymphadenopathy, splenomegaly, and cytopenias. Hemophagocytosis and elevated sCD25 have also been reported in ALPS, which overlap with the clinical features of HLH (9, 10) and make the distinction between ALPS and HLH nebulous.

**Figure 3:**
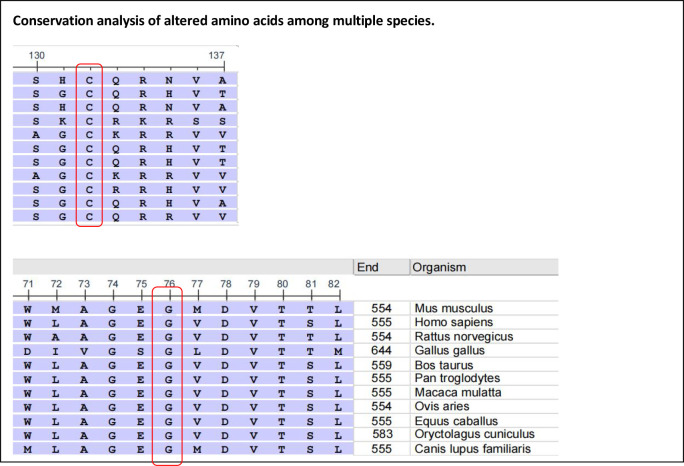
Alignment of the human perforin protein with that of other species. Red boxes highlight G47 (left) and C102 (right), respectively.

Perforin protein has three domains, among which the membrane attack complex/perforin superfamily (MACPF)/CDC domain enables perforin to polymerize in the target cell membrane to form pores. This facilitates the entry of granzyme and other dissolved molecules, released by cytotoxic T lymphocytes (CTLs) and NK cells, into the cytoplasm to induce apoptosis. The *PRF1* variants c.139G>T (p.G47C) and c.305G>T (p.C102F) have been previously reported as pathogenic mutations (11–13). These mutations are located in the MACPF domain of the perforin protein. Through bioinformatics approaches, the authors suggested that the mutations may affect the stability or oligomerization of the MACPF domain. Phylogenetic analysis revealed that G47 and C102 are highly conserved among vertebrates (**Figure 3**), suggesting that these two sites play important roles in the structure and function of the perforin protein. The predicted structure of the perforin p.C102F mutant showed a loss of disulfide bonds, which might affect the stability of the perforin protein. No changes in hydrogen bonds or disulfide bonds were found between the wild-type perforin and the perforin p.G47C mutant, indicating the structural stability of the protein. However, several gene mutation prediction software programs predict it to be harmful. Moreover, the patient’s mother and elder sister harbored the p.G47C variant in the heterozygous state, and his father harbored the p.C102F variant in the heterozygous state; all of them were healthy. This finding is consistent with what was reported in previous studies (14). One possibility is that the compound heterozygous mutation resulted in the development of HLH in the child. Alternatively, other contributing factors, such as additional genetic variations, may have also played a role in the onset of HLH.

It is well known that the activation of CD8^+^ T lymphocytes is central to the pathophysiology of FHL. In a normal immune response, triggering antigen clearance leads to the apoptosis of most antigen-specific effector cells, thereby preventing ongoing inflammation. In the case of defective cytotoxicity, these cells cannot be cleared, which perpetuates the cycle of hyperinflammation. Therefore, other factors influencing this mechanism may also promote the occurrence of HLH. Some researchers have conducted whole-exome sequencing analysis of FHL family members to identify the potential “second-hit” mechanism underlying the onset of FHL2. The patient’s brother carried the same compound heterozygous mutations in both PRF1 alleles and had an EBV infection, but he remained healthy. In the proband, a homozygous missense mutation (S1006L) was identified in the *PCDH18* gene. This mutation (S1006L) could lessen the *PCDH18*-induced inhibition of target cell activation and reduce the apoptosis of T lymphocytes. These findings suggest that this mutation might represent a potentially critical mechanism underlying the onset of FHL2. In our patient, an additional spontaneous somatic mutation in *FAS* (c.776T>C) was detected in addition to the mutation in *PRF1*. The *FAS* pathway plays an important role in mediating T-lymphocyte apoptosis and participates in CTL-mediated cytotoxicity. Mutations in the *FAS* gene can lead to the inhibition of Fas/FasL-mediated apoptosis, which in turn leads to the accumulation of activated T lymphocytes. Therefore, we speculated that the *FAS* mutation may be the “second hit” for the onset of HLH in this patient.

Fas-deficient T lymphocytes in patients from ALPS can mediate apoptosis through cytotoxic granule-dependent pathways. An increased number of circulating T lymphocytes expressing granzymes A and B has been detected (15). In the context of Fas deficiency, the latter pathway may compensate for Fas defects to some extent. This might explain why variations in perforin and UNC13D, which are involved in the secretion or exocytosis of granzymes, are risk factors for ALPS development.

Both the Fas/FasL pathway and the cytolytic granule pathway can mediate the apoptosis of activated T lymphocytes. In the case of defects in both pathways, activated lymphocytes accumulate more, and virus-infected cells are less able to be cleared. In mice, defects in perforin and Fas-dependent killing of dendritic cells (DCs) may lead to chronic activation of CD8^+^ T cells, profoundly increased levels of IFN-γ, increased lymphocytic infiltration into organs, increased severity of pancytopenia, and early lethality (16). Boggio et al. (17) described a case of a child with *FAS*, *UNC13D*, and *XIAP* mutations, whose clinical manifestations were mixed with the characteristics of ALPS, FHL, and XLP. Fever without bacterial infection occurred at 12 years of age, with suspected macrophage activation and, soon, death. It has been reported that in T/NK lymphoma patients, *FAS* mutations may increase the risk of HLH, with a very high mortality rate (18). Similarly, in our patient who carried *FAS* and *PRF1* mutations and presented with ALPS and FHL characteristics, the disease was aggressive, with early death. The perforin/granzyme system and Fas/FasL have synergistic effects on the cytotoxicity and apoptosis of activated T lymphocytes and NK cells. These synergies may explain the overlapping clinical manifestations of the two diseases in the patient, while also leading to more pronounced cell accumulation, excessive inflammatory responses, and adverse outcomes.

Owing to genetic variations, patients with FHL still have a lifelong risk of disease recurrence even if they achieve remission after conventional treatment. Reconstructing the defective immune system through allo-hematopoietic stem cell transplantation (HSCT) is the only way to cure FHL. At present, multiple studies have confirmed that the most important factor affecting overall survival (OS) and event-free survival (EFS) in FHL patients treated with HSCT is remission status before HSCT (19). For patients whose disease cannot be controlled by conventional treatment, selecting second-line or salvage treatment regimens before transplantation has become the key to survival after transplantation. However, Messina et al. (20) reported on 109 patients with HLH who underwent HSCT and stated that the presence of active disease at transplantation did not significantly affect prognosis. In contrast, postponing HSCT could expose the patient to the risk of deterioration of general status, thus worsening the transplant outcome. Therefore, for patients with active disease that cannot be controlled by front- or second-line therapy, performing allo-HSCT as early as possible may be beneficial. For the patient described in this case, given the difficulty in achieving remission of HLH, early HSCT may have been the critical intervention for his survival.

## Conclusion

In summary, this case involving a child with mutations in the *PRF1* and *FAS* genes presented with both HLH and ALPS phenotypes and deteriorated rapidly, resulting in a poor prognosis. The *FAS* mutation may represent the “second hit” that triggered the onset of FHL. The comutation of the FAS and *PRF1* may have contributed to the adverse outcome in this patient. Thus, clinicians should be aware of the impact of this comorbidity when developing strategies to treat HLH, and should consider allogeneic hematopoietic stem cell transplantation at an early stage if conditions permit.

## Data Availability

The original contributions presented in the study are included in the article/supplementary material. Further inquiries can be directed to the corresponding authors.
